# Collaboration between a cis-interacting natural killer cell receptor and membrane sphingolipid is critical for the phagocyte function

**DOI:** 10.3389/fimmu.2024.1401294

**Published:** 2024-04-24

**Authors:** Hitomi Karyu, Takahiro Niki, Yuriko Sorimachi, Shoji Hata, Shiho Shimabukuro-Demoto, Tetsuya Hirabayashi, Kojiro Mukai, Kohji Kasahara, Keiyo Takubo, Nobuhito Goda, Koichi Honke, Tomohiko Taguchi, Hiroyuki Sorimachi, Noriko Toyama-Sorimachi

**Affiliations:** ^1^ Division of Human Immunology, International Vaccine Design Center, The Institute of Medical Science, The University of Tokyo (IMSUT), Tokyo, Japan; ^2^ Laboratory for Neural Cell Dynamics, RIKEN Center for Brain Science, Saitama, Japan; ^3^ Department of Life Science and Medical Bioscience, School of Advanced Science and Engineering, Waseda University, Tokyo, Japan; ^4^ Department of Stem Cell Biology, Research Institute, National Center for Global Health and Medicine, Shinjuku, Tokyo, Japan; ^5^ Tokyo Metropolitan Institute of Medical Science, Tokyo, Japan; ^6^ Laboratory of Biomembrane, Tokyo Metropolitan Institute of Medical Science, Tokyo, Japan; ^7^ Department of Integrative Life Sciences, Graduate School of Life Sciences, Tohoku University, Sendai, Japan; ^8^ Department of Biochemistry and Kochi System Glycobiology Center, Kochi University Medical School, Kochi, Japan

**Keywords:** inhibitory receptor, Ly49, MHC class I, cis interaction, sphingolipids, 2 phagocytosis, lipid raft, macrophage

## Abstract

Inhibitory natural killer (NK) cell receptors recognize MHC class I (MHC-I) in *trans* on target cells and suppress cytotoxicity. Some NK cell receptors recognize MHC-I in *cis*, but the role of this interaction is uncertain. Ly49Q, an atypical Ly49 receptor expressed in non-NK cells, binds MHC-I in *cis* and mediates chemotaxis of neutrophils and type I interferon production by plasmacytoid dendritic cells. We identified a lipid-binding motif in the juxtamembrane region of Ly49Q and found that Ly49Q organized functional membrane domains comprising sphingolipids via sulfatide binding. Ly49Q recruited actin-remodeling molecules to an immunoreceptor tyrosine-based inhibitory motif, which enabled the sphingolipid-enriched membrane domain to mediate complicated actin remodeling at the lamellipodia and phagosome membranes during phagocytosis. Thus, Ly49Q facilitates integrative regulation of proteins and lipid species to construct a cell type-specific membrane platform. Other Ly49 members possess lipid binding motifs; therefore, membrane platform organization may be a primary role of some NK cell receptors.

## Introduction

Natural killer (NK) cells become tolerant to healthy self cells by sensing the presence of self MHC class I (MHC-I) (1, 2). In self and nonself discrimination, MHC-I interacts with its receptors in *trans*; that is, receptors bind to ligands expressed on other cells. In addition, a number of receptors interact with MHC-I expressed on the same cell, i.e. a *cis* interaction (3), which seems to be not involved in self and nonself discrimination. Some *cis-*interacting receptors have immune regulatory functions (4), but their roles remain largely unknown.

A type II membrane spanning receptor bearing an immunoreceptor tyrosine-based inhibitory motif (ITIM), Ly49Q, is a member of the NK cell receptor family (5–7). However, this receptor is expressed not on NK cells but rather on plasmacytoid dendritic cells (pDCs), neutrophils, and macrophages (Mϕs) (7). Similar to other NK cell receptors, Ly49Q recognizes MHC-I molecules, and the association between Ly49Q and MHC-I occurs in a *cis* configuration (8). We previously demonstrated that this molecule is critical for type I interferon (IFN-I) production by pDCs (9–12) and for the polarization and chemotaxis of neutrophils (13). Ly49Q colocalizes with the ganglioside GM1 and contributes to lipid raft endocytosis and endolysosomal trafficking (13). Furthermore, Ly49Q plays a unique role as a switch device to induce tonic responses in innate immune cells; that is, Ly49Q functions as an inhibitory receptor in the steady state, but once cells receive inflammatory stimuli, it behaves like an activation receptor by controlling Src family kinases (SFK) and PI3K activation (13).

Mϕs are professional scavengers and can efficiently engulf relatively large particulate materials, such as microbes and apoptotic cells (14, 15). Phagocytosis, which is defined by the ability to engulf particulates exceeding 0.5 µm in diameter, is mediated by phagocytic receptors (15). A broad spectrum of phagocytic receptors, including Fcγ receptors (FcγRs) and lectins, have been identified, and their phagocytic mechanisms have been investigated. Although phagocytic receptors show structural variations and differentially activate membrane-proximal signaling molecules, the common events in phagocytosis are actin remodeling and endocytosis (16). Actin remodeling during phagocytosis is a complex and sequentially regulated process that is a continuation of pseudopod formation. Pseudopods probe for and envelop prey, and the nascent phagocytic vacuole undergoes maturation in which the strict regulation of periphagosomal actin is important. Mϕs can engulf particles over 20 µm in diameter despite their own diameter being approximately 14 µm in size (17). The dynamic change in membrane capacity in focal exocytosis events is concurrently processed with actin remodeling during phagocytosis (18). Therefore, it is important to understand how lipid environments and membrane behaviors are coordinately regulated by actin remodeling in phagocytosis.

The signaling events during FcγR-mediated phagocytosis that lead to plasma membrane pseudopod extension and particle engulfment have been largely elucidated, and the critical roles of Rho GTPases, such as Rac1/2, Cdc42 and RhoA, in shaping and regulating the dynamics of F-actin have been established (16). Strict regulation of phosphoinositide species by phosphoinositide (PI) kinases and their phosphatases, such as Src homology 2 (SH2) domain containing inositol polyphosphate 5-phosphatase 1 (SHIP1) and SHIP2, are at the core of actin regulation (19, 20). In addition to these mechanisms, cholesterol in the plasma membrane is essential for phagocytosis because cholesterol depletion by methyl-β-cyclodextrin (mβCD) severely diminishes phagocytosis (21). Although cholesterol depletion is a harsh treatment, it is believed that membrane lipid microdomains, often referred to as lipid rafts, are needed for phagocytosis. However, a comprehensive understanding of membrane proteins, such as phagocyte receptors, and their interaction with peripheral membrane lipids, including their contribution to lipid rafts, is lacking.

Lipid rafts are defined as cholesterol- and sphingolipid-rich membrane domains and are considered to be important signaling platforms (22–24). Cholesterol and sphingolipids possess saturated fatty acyl chains and organize into tightly packed, liquid-ordered membrane microdomains (25). Various cell surface receptors and intracellular signaling molecules, such as glycosylphosphatidylinositol (GPI)-anchored proteins, Src family kinases (SFK) and small GTPases, are associated with lipid rafts, and such raft-associated proteins are frequently modified with lipid moieties to markedly increase their hydrophobicity (22, 23, 25). Although the significance of partitioning proteins and lipids into a specialized membrane domain is generally accepted, there has been much debate concerning the definition, size, and experimental adequacy of lipid rafts (26). There are several outstanding questions in the field of rafts and functional lipid microdomains. For example, what is the mechanism that regulates raft formation, how are raft properties determined, how is the movement and trafficking of raft domains regulated, and how does the regulation of raft assembly and movement contribute to cellular responses? Elegant studies using liposomes have shown the self-assembly of cholesterols and sphingolipids to form liquid-ordered domains (27–29). These observations strongly indicate that, in cell membranes, including plasma and organelle membranes, membrane domains are arranged to some extent depending on the lipid environment.

In the present study, we identified the cholesterol recognition/interaction amino acid consensus sequence (CRAC) motifs (30, 31) at lipid membrane boundary regions of Ly49Q and other Ly49 molecules and demonstrated the Ly49Q association with sulfatide via its CRAC motif. Our findings revealed a collaborative function between a previously known ITIM and a newly identified CRAC motif of Ly49Q in phagocytosis, which may be helpful in considering the role of cis-interactions of other NK cell receptors expressed on NK cells.

## Results

### Ly49Q is necessary for FcγR-mediated phagocytosis in Mϕs

**Figure 1:**
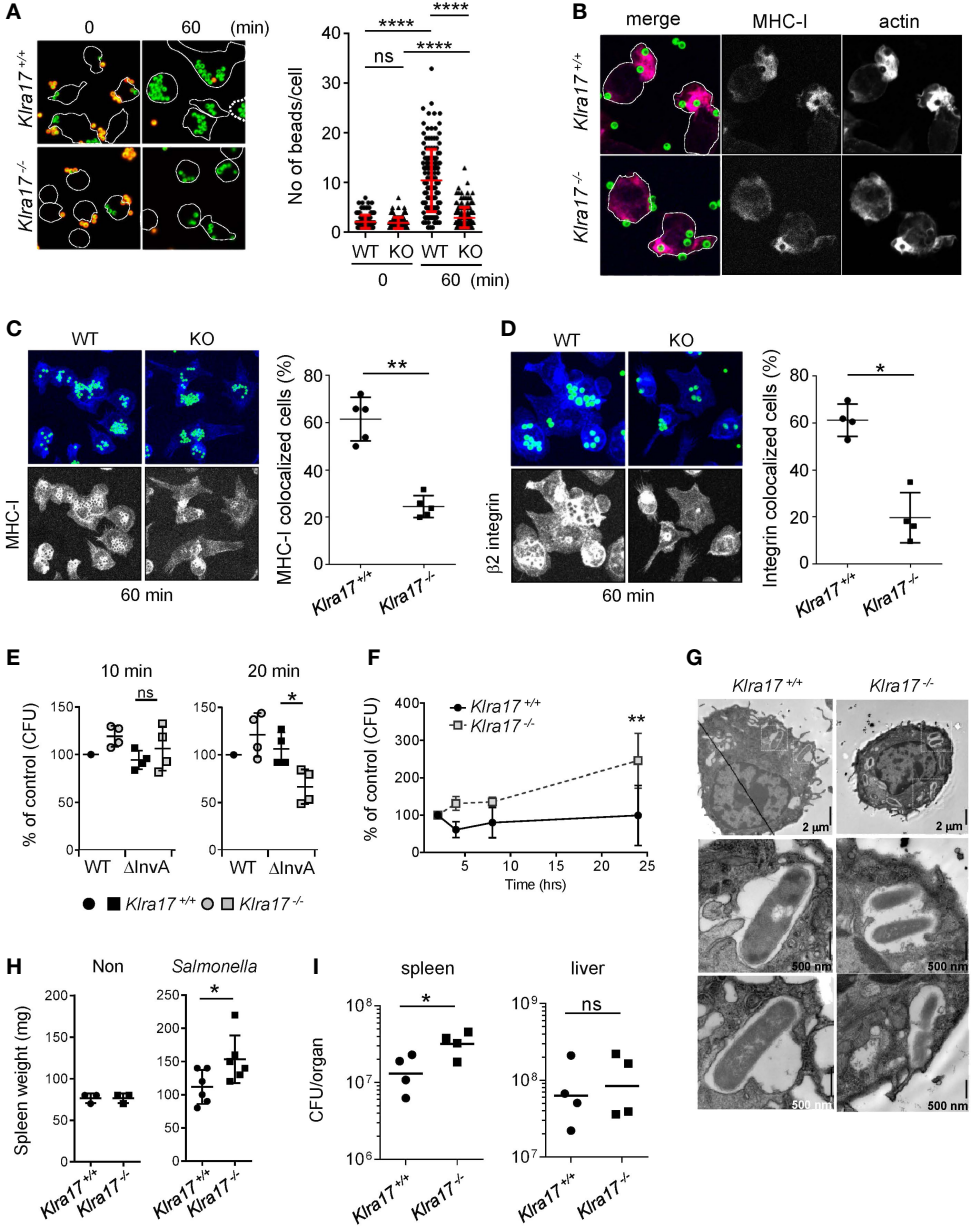
Ly49Q is needed for Mϕ phagocytosis and protection from *Salmonella* infection. **(A)** Phagocytosis of IgG-opsonized, fluorescence-labeled, 2-µm-diameter latex beads by PEMϕ. Surface-bound beads were discriminated by staining with Alexa594-conjugated anti-mouse IgG antibody without cell permeabilization. The numbers of green fluorescent beads within a cell were counted, and more than 25 cells in each sample were analyzed. **(B)** Immunofluorescence images showing colocalization of MHC-I and F-actin in PEMϕs after incubation with fluorescence-conjugated latex beads (ϕ2 µm). **(C, D)** Immunofluorescence staining of MHC-I **(C)** and β2 integrin **(D)** 60 min after latex beads were added (left). Five nonoverlapping pictures including more than 30 cells were randomly taken, and the frequency of cells showing colocalization of MHC-I with beads was determined (right). MHC-I and β2 integrin were visualized by Alexa647-conjugated secondary anti-rat IgG antibody. In these experiments, unlike A and B above, opsonized beads were not stained with Alexa594-conjugated anti-mouse IgG antibody. The merged area of the beads (FITC) and MHC-I/β2 integrin (Alexa647) was detected as light blue, while MHC-I and B2 integrin were detected as blue, with blue accumulation at the bottom and around the beads. To better visualize the localization of MHC-I and β2 integrin, the blue region was extracted and displayed in black and white contrast. **(E)** Attachment and invasion of WT and noninvasive Salmonella Tiphi (ΔinvA) into PEMϕs was examined as described in Methods. **(F)** Survival and proliferation of Salmonella Tiphi were followed for 24 hours after infection. **(G)** Electron microscopy analyses of Salmonella ingested by PEMϕ. White boxes in the upper photographs indicate areas enlarged in the bottom images. **(H)** Weights of spleens before and after Salmonella infection. **(I)** Bacterial titers in the spleens and livers were determined for mice infected with *Salmonella*. CFU; colony-forming units. Data points represent organs from a single mouse. Statistical analyses were conducted using Mann–Whitney U test. ****P<0.0001; **P<0.01; *P<0.05; ns, not significant. All results shown are representative of at least two separate experiments.

We examined the role of Ly49Q (encoded by *Klra17* gene) in FcγR-mediated phagocytosis. *Klra17^-/-^* peritoneal Mϕs (PEMϕs) ingested fewer beads than *Klra17^+/+^* PEMϕs (**Figure 1A**). *Klra17^+/+^* PEMϕs formed large, directional membrane protrusions promptly after bead binding (**Figure 1B**). Conversely, *Klra17^-/-^* PEMϕs did not exhibit membrane ruffling, although F-actin colocalized with beads (**Figure 1B**). Similarly, impaired membrane ruffling was also observed in *Klra17^-/-^* PEMϕs by phorbol ester stimulation, which mimics the activation of PKC downstream of FcγR (**Supplementary Figures 1A, B, Supplementary Movies 1**, **2**). These results indicated that Ly49Q increases FcγR-mediated phagocytosis, possibly by mediating the formation of ruffled membranes.

After incubation for 60 min, the accumulation of MHC-I around phagosomes was observed in both *Klra17^+/+^* and *Klra17^-/-^* PEMϕs, although it was less efficient in *Klra17^-/-^* PEMϕs than in *Klra17^+/+^* ones (**Figure 1C**). β2 integrins, which are needed for optimal phagocytosis (32), were recruited to bead-containing phagosomes in *Klra17^+/+^* PEMϕs, but β2 integrins in *Klra17^-/-^* PEMϕs localized to different regions from bead-containing phagosomes (**Figure 1D**). These results indicated that Ly49Q affected not only phagocytosis efficiency but also phagosome membrane properties by recruiting membrane proteins like MHC-I and β2 integrins.

### Ly49Q-dependent properties of phagocytes are important for protection against Salmonellae

We next confirmed the significance of Ly49Q in pathogen engulfment. *Salmonella enterica* serovar *Typhimurium* SH100 (*S. typhi* WT) is a Gram-negative bacterium that infects host cells using the type III secretion system (T3SS) (33). To distinguish phagocytosis from infection, we used the virulence-defective strain *S. typhimurium* ΔinvA (*S. typhi* ΔinvA), which is an invasion-defective strain and is thus ingested by phagocytosis (34). Bacterial attachment (after 10 min incubation) did not differ markedly between *S. typhi* WT and *S. typhi* ΔinvA strains or between *Klra17^+/+^* and *Klra17^-/-^* PEMϕs (**Figure 1E**). Phagocytosis of the opsonized *S. typhi* ΔinvA strain was decreased in *Klra17^-/-^* PEMϕs compared with *Klra17^+/+^* PEMϕ,s, although T3SS-mediated infection of *S. typhi* WT strains did not markedly differ between *Klra17^+/+^* and *Klra17^-/-^* PEMϕs (**Figure 1E**). Unexpectedly, the bactericidicity of *Klra17^-/-^* PEMϕ against virulent *S. typhi* WT was significantly decreased (**Figure 1F**). Activation of ERK and NF-κB by *S. typhi* WT infection was impaired in *Klra17^-/-^* PEMϕs (**Supplementary Figure 1C**) (35, 36); consequently, IL6 production was diminished in *Klra17^-/-^* PEMϕs (**Supplementary Figure 1D**). An electron microscopic analysis revealed that although both *Klra17^+/+^* and *Klra17^-/-^* PEMϕs showed *Salmonella*-containing vesicles (SCVs), those in *Klra17^-/-^* PEMϕs contained multiple *Salmonella* particles (**Figure 1G**) (37), possibly due to reduced bactericidal activity. *In vivo* infection experiments demonstrated that *Klra17^-/-^* mice showed augmented splenomegaly and increased titers of *S. typhi* in the spleen (**Figures 1H, I**). These results indicated that Ly49Q plays a role not only in the phagocytosis of opsonized bacteria but also in the bactericidal activity both *in vitro* and *in vivo*.

### The Ly49Q ITIM mediates membrane ruffling in the phagocytic region

**Figure 2:**
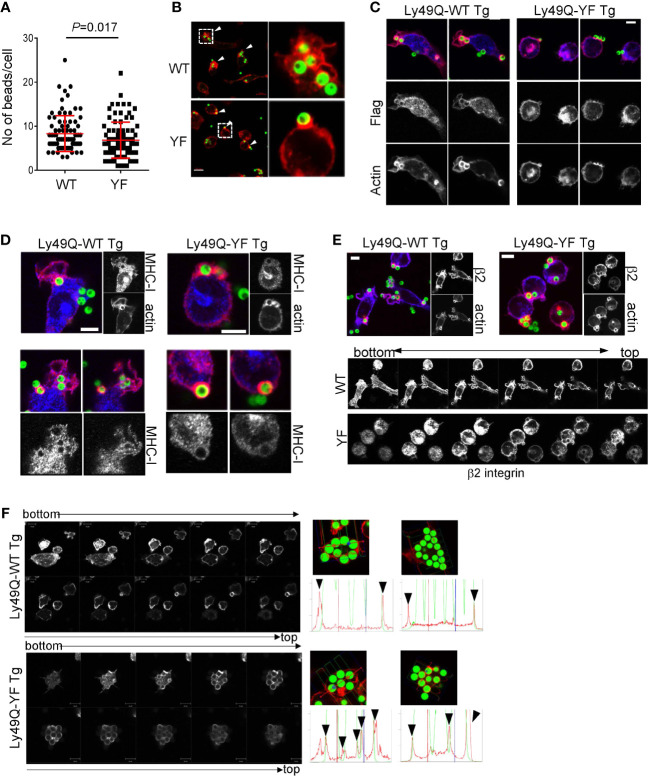
Phagocytosis of IgG-opsonized latex beads in PEMϕs expressing Ly49Q-WT or Ly49Q-YF. **(A)** The numbers of 6-µm-diameter phagocytosed beads were counted. Statistical analyses were conducted using Mann–Whitney U test. **(B)** Immunohistochemical analyses of actin structures in PEMϕs soon after the addition and centrifugation of fluorescence-labeled beads (ϕ2 µm). Arrow heads indicate the sites where beads bound and actin polymerized. White boxes in the left photographs indicate the areas enlarged in the right photographs. Scale bar; 10 µm. **(C)** Localization of Ly49Q visualized by anti-Flag staining. PEMϕ prepared from Ly49Q-WT or Ly49Q-YF Tg mice were fixed soon after the addition and centrifugation of fluorescence-labeled beads (ϕ2 µm) and stained with the reagent indicated. **(D, E)** Distribution of MHC-I **(D)** and β2 integrin **(E)** in PEMϕ after capturing latex beads (ϕ2 µm). Lower pictures in **(E)** show serial sections of Z-planes of the PEMϕs stained with anti-β2 integrin-antibody shown in the upper images. **(F)** F-actin distribution in PEMϕs 60 min after bead addition. Serial sections of the Z-plane of F-actin-visualized PEMϕs are shown on the left. Histograms on the right show the profiles of signal intensities along the dashed lines in the photographs. All results shown are representative of at least two independent experiments.

We next examined the role of Ly49Q ITIM in phagocytosis using PEMϕs obtained from ITIM-less Ly49Q (Ly49Q-YF) transgenic *Klra17^-/-^* mice. The number of 2-µm-diameter ingested beads was not significantly affected by the presence or absence of an ITIM (**Supplementary Figures 2A, B**). The number of 6-µm-diameter ingested beads was reduced in the Ly49Q-YF PEMϕs compared with the Ly49Q-WT PEMϕs (**Figure 2A**). However, the degree of reduced phagocytosis caused by ITIM deficiency is less than that caused by Ly49Q deficiency (**Figure 1A**), indicating that ITIM contributes to the efficiency of FcγR-mediated phagocytosis but is not indispensable for this type of phagocytosis. However, we found that the ITIM of Ly49Q was important for actin remodeling in membrane regions where phagocytosis occurs. Ly49Q-WT PEMϕs showed membrane ruffles wrapping around multiple beads (**Figure 2B**). In contrast, in Ly49Q-YF PEMsϕ, beads were captured on cup-like F-actin structures without membrane ruffling (**Figure 2B**). These results indicated that the Ly49Q ITIM is needed for actin remodeling to make membrane ruffles at the site of phagocytosis. Ly49Q-WT proteins were detected around beads and membrane ruffles, but Ly49Q-YF was predominantly located in intracellular vesicular compartments, although it was weakly detected in the region of bead attachment (**Figure 2C**), indicating that the distribution of Ly49Q is regulated in an ITIM-dependent manner. Furthermore, ITIM was needed for the recruitment of MHC-I and β2 integrin at phagosomes (**Figures 2D, E**). Collectively, these results indicated that the ITIM of Ly49Q modifies the mode of phagocytosis of Mϕs by forming ruffled membranes and mobilizing integrins to phagosomes.

### The Ly49Q ITIM controls actin remodeling in phagosomes

Since ruffled and nonruffled membranes are sources that provide phagosomes with lipid membranes with different lipid components and proteins (16, 38, 39), we hypothesized that Ly49Q ITIM might affect phagosome properties. Immunohistochemical analyses revealed that, in Ly49Q-YF PEMϕs, phagosomes containing ingested beads were individually surrounded by polymerized actin, while in Ly49Q-WT PEMsϕ, multiple beads were surrounded together by F-actin (**Figure 2F**), resembling macropinocytosis (39). These results suggested that Ly49Q modulates actin remodeling not only during phagocytosis but also in subsequent phagosome formation in an ITIM-dependent manner.

Actin polymerization around a nascent phagosome is transient, and only immature phagosomes possess an actin coat assembly, which greatly influences phagosome maturation by contributing to vesicle docking, fusion, and trafficking (40–42). Therefore, altered properties of phagosomes (**Figures 1C, D**) and SCV (**Figure 1G**) in *Klra17^-/-^* PEMϕs may be associated with differences in the particle uptake manner, including actin remodeling.

### MHC-I is also needed for membrane ruffle formation and actin remodeling in phagocytosis

**Figure 3:**
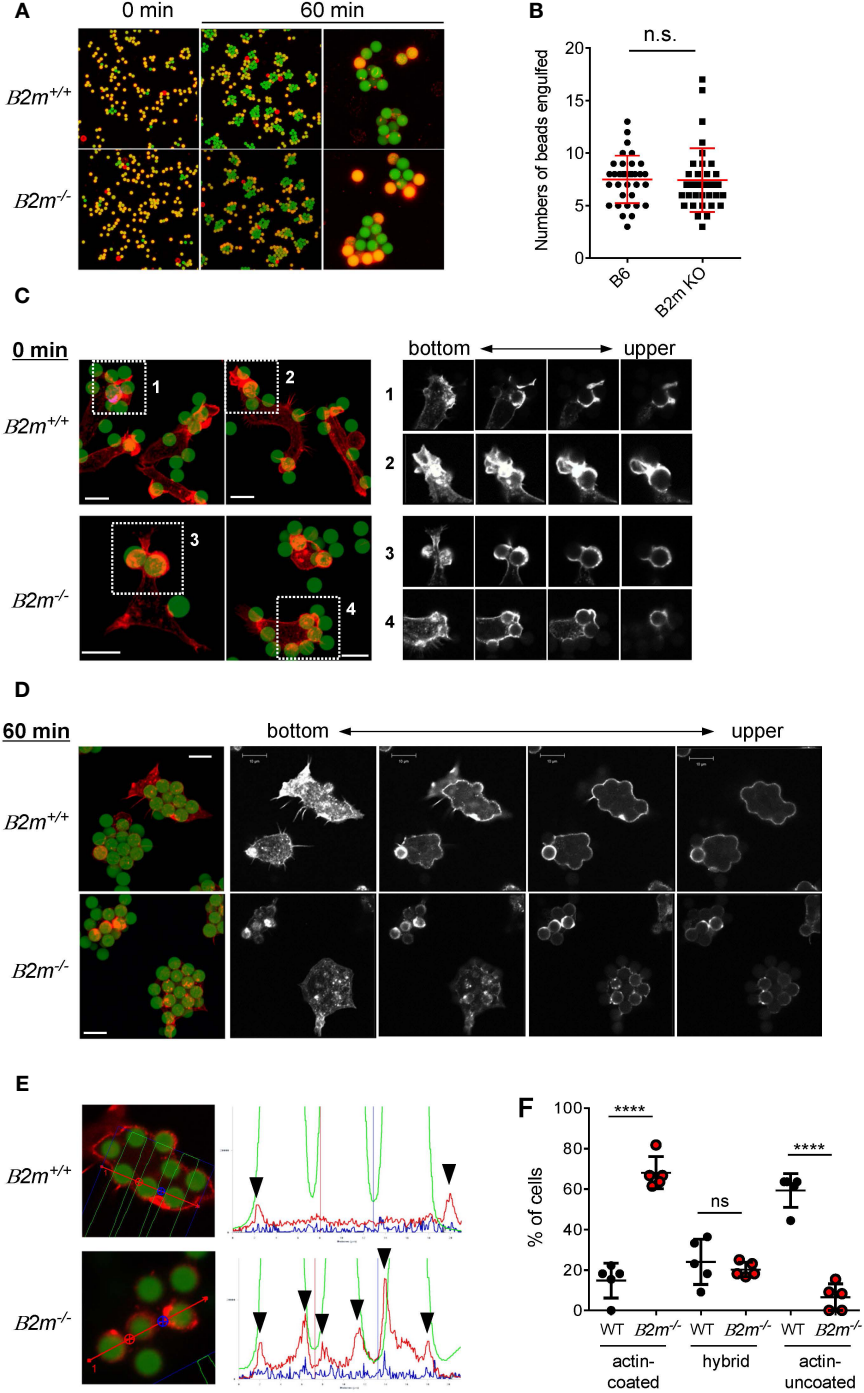
Phagocytosis of IgG-opsonized latex beads in *B2m* ^-/-^ PEMϕ. **(A)** Fluorescence microscopy view of PEMϕs incubated with 6-µm-diameter IgG-opsonized beads for the indicated periods. Surface-bound beads were discriminated by staining with Alexa Fluor 594-conjugated anti-mouse IgG antibody without permeabilization. **(B)** The numbers of phagocytosed beads were counted. **(C)** Fluorescence micrograph of PEMϕ with FITC beads attached (left). PEMϕs were fixed soon after the beads were attached by centrifugation. F-actin was visualized with Alexa592-conjugated phalloidin. White boxes in the left images indicate enlarged areas shown as serial sections in the z-plane (right). Red-stained regions were extracted and displayed in black and white contrast to better visualize the localization of polymerized actin. **(D)** Fluorescence micrograph of PEMϕ-ingested beads (left) and serial sections in the Z-plane of F-actin staining of PEMϕs with Alexa592-conjugated phalloidin (right) after incubation for 60 min. Red-stained regions were extracted and displayed in black and white contrast to better visualize the localization of polymerized actin. **(E)** Histograms at right show signal intensity profiles of beads (green) and F-actin (red) along the dashed lines in the photographs at left. **(F)** Frequencies of cells possessing phagosomes with and without an actin coat. Hybrid represents cells possessing both actin-coated and actin-uncoated phagosomes. All results shown are representative of at least two independent experiments. Statistical analyses were conducted using Mann–Whitney U test **(B, F)**. ****P<0.0001; ns, not significant.

We next examined whether or not the binding partner of Ly49Q, MHC-I, contributes to Mϕ phagocytosis. PEMϕs obtained from β2-microglobulin deficient (*B2m* ^-/-^) mice, in which MHC-I expression is greatly decreased (43), engulfed IgG-opsonized beads, and there was no significant difference in the number of beads phagocytosed by *B2m* ^-/-^ and *B2m* ^+/+^ PEMϕs, regardless of particle size (**Figures 3A, B**). However, membrane ruffling and actin remodeling in *B2m* ^-/-^ PEMϕs were phenocopies of those in Ly49Q-YF PEMϕs in membrane regions where phagocytosis occurs; *B2m* ^+/+^ PEMϕs promptly formed membrane ruffles around beads, whereas *B2m* ^-/-^ PEMϕs did not, although polymerized actin surrounded the beads in both PEMϕs (**Figure 3C**). Although both *B2m* ^+/+^ and *B2m* ^-/-^ PEMϕs engulfed multiple beads in their cytoplasm, periphagosomal F-actin was lost in *B2m* ^+/+^ but not in *B2m* ^-/-^ PEMϕs at the indicated time points (**Figures 3D, E**). The frequency of cells showing F-actin-uncoated phagosomes was significantly higher in *B2m* ^+/+^ PEMϕs than in *B2m* ^-/-^ PEMϕs (**Figure 3F**). Conversely, the frequency of cells showing F-actin-coated phagosomes was significantly higher in *B2m* ^-/-^ PEMϕs than in *B2m* ^+/+^ PEMϕs (**Figure 3F**). The frequency of hybrid-type cells showing both F-actin-uncoated and F-actin-coated phagosomes was not affected by the presence or absence of β2-microglobulin (**Figure 3F**).

Taken together, these findings suggest that MHC-I is involved in determining the mode of FcγR-mediated phagocytosis and in phagosome destiny. Based on the *cis*-interaction between Ly49Q and MHC-I, these findings imply that the role of MHC-I in actin remodeling during phagocytosis is exerted via the interaction with Ly49Q.

### The Ly49Q ITIM recruits signaling molecules for actin remodeling

Inhibitory NK cell receptors with ITIM interact with MHC-I and are considered to induce tyrosine phosphorylation of ITIM. Phosphorylated ITIM recruits SHP1 and SHP2, which is also the case for the Ly49Q ITIM (13). However, it seemed difficult to explain the complex actin remodeling associated with phagocytosis solely by the function of these protein phosphatases. Therefore, we further exhaustively searched for ITIM-associated molecules using a synthetic peptide corresponding to a phosphorylated ITIM (pITIM) of Ly49Q.

**Figure 4:**
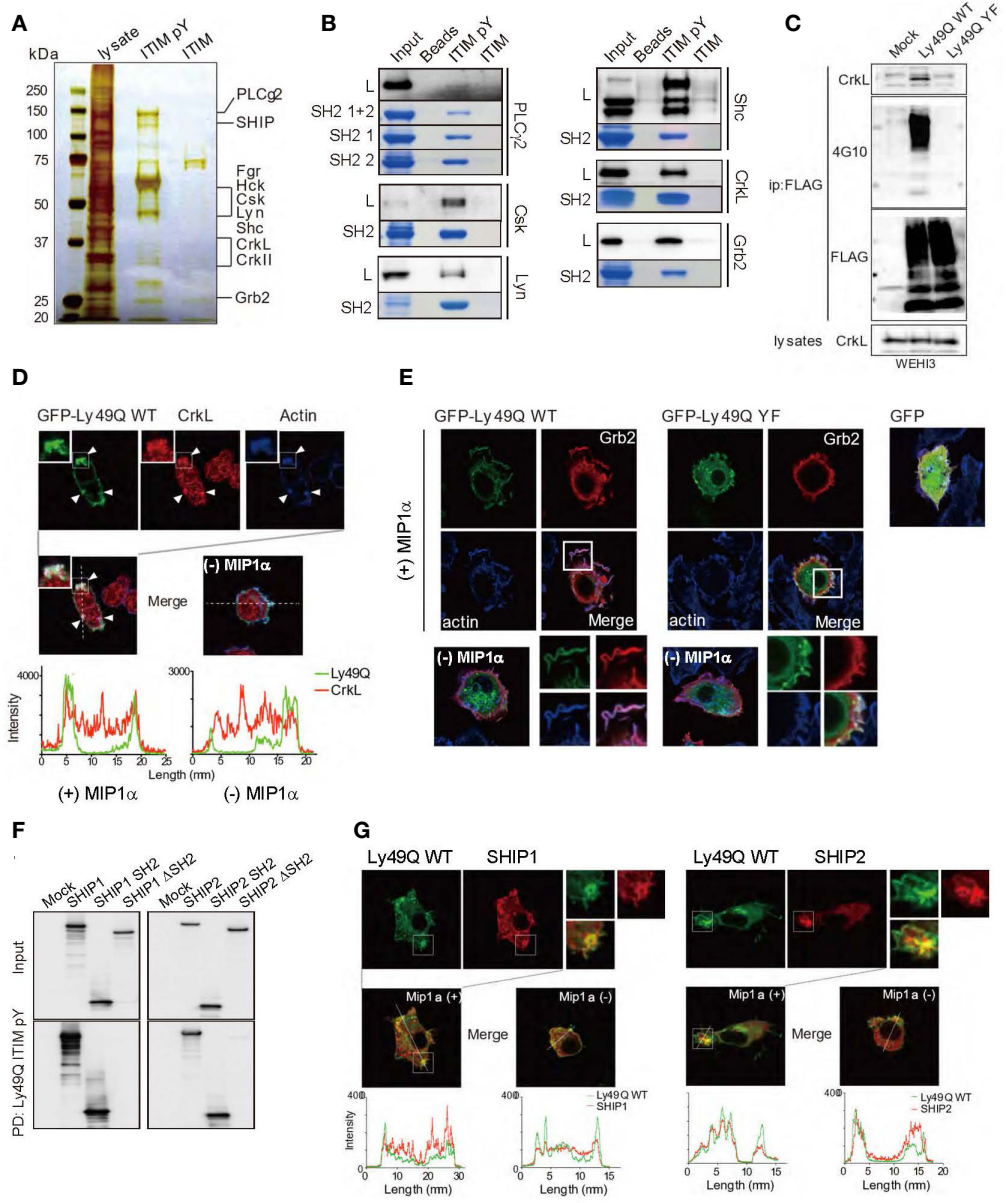
Identification of Ly49Q ITIM-interacting proteins. **(A)** Proteins associated with peptides corresponding to the tyrosine-phosphorylated (ITIM pY) or nonphosphorylated (ITIM) ITIM of Ly49Q were analyzed by SDS−PAGE and visualized by silver staining. **(B)** Association of the identified proteins with ITIM pY was confirmed by peptide pull-down assays. Raw264.7 cell lysates (L) or the recombinant SH2 domains of the identified proteins (SH2 or SH2 1 + 2) were incubated with the indicated peptide-conjugated resins and analyzed by SDS−PAGE. The bound endogenous proteins pulled down from RAW264.7 cell lysates (black bands) and the recombinant SH2 domain fragments pulled down from *E. coli* soluble fractions (blue bands) were visualized by immunoblotting and Coomassie brilliant blue staining, respectively. **(C)** Ly49Q was precipitated using anti-FLAG antibodies from the cell lysates of pervanadate-treated WEHI3 transfectants expressing FLAG-tagged Ly49Q-WT or Ly49Q-YF. The precipitates were analyzed by western blotting. The results indicated that Ly49Q could recruit endogenous CrkL in an ITIM tyrosine phosphorylation-dependent manner. **(D, E)** Immunofluorescence images showing colocalization of Ly49Q with CrkL **(D)** and Grb2 **(E)**. RAW264.7 cells were transfected with plasmids encoding GFP-tagged Ly49Q-WT or Ly49Q-YF, and GFP fluorescence (green) and endogenous CrkL (red in D) or Grb2 (red in E) were analyzed by confocal microscopy. Boxed regions are enlarged at the bottom right side. The distributions of these molecules in the absence of MIP1α are shown in the photographs on the bottom left side. Histograms in **(D)** show the fluorescence intensity profiles along the dashed lines in the upper photographs. Arrowheads indicate sites of colocalization of Ly49Q, CrkL, and polymerized actin. **(F)** Cell lysates of HEK293T cells transfected with 6× Myc-tagged forms of SHIP, SHIP lacking the SH2 domain, or the SH2 domain of SHIP as indicated were subjected to peptide pull-down assays with ITIM pY. The precipitates were analyzed by immunoblotting using an anti-Myc antibody. **(G)** Immunofluorescence images showing the colocalization of Ly49Q with SHIP1 (left) or SHIP2 (right). RAW264.7 cells were transfected with plasmids encoding GFP-tagged Ly49Q-WT together with Myc-tagged SHIP1 or Myc-tagged SHIP2, and GFP fluorescence (green) and anti-Myc antibody-stained SHIP1 (red) were analyzed by confocal microscopy. Boxed regions are enlarged at the middle left side. On the middle right side, images of cells in the absence of Mip1α are shown. Histograms at the bottom show the fluorescence intensity profile along the dashed lines in the upper photographs. All results shown are representative of at least two independent experiments.

Multiple proteins that selectively associated with pITIM but not with nonphosphorylated ITIM were identified by mass spectrometry (MS) (**Figure 4A**). The direct association between the pITIM and SH2 domains of the identified proteins was confirmed using recombinant proteins *in vitro* (**Figure 4B**), indicating that Ly49Q has the potential to associate with multiple molecules in addition to protein phosphatases. Among the molecules that bind to pITIM, we focused on the SH2/SH3-containing adaptor proteins Grb2 and CrkL, both of which are involved in actin remodeling by interacting with guanine nucleotide exchange factors (GEFs), such as Sos and C3G, that couple with Rho family members (44–46). CrkL was detected in the Ly49Q immunoprecipitates in an ITIM-dependent manner (**Figure 4C**). Both Grb2 and CrkL colocalized with Ly49Q at chemokine-induced ruffled membranes in RAW264.7 cells (**Figures 4D, E**). Ly49Q-YF also partially colocalized with Grb2, but chemokine-induced membrane ruffling was not observed in RAW264.7 cells expressing Ly49Q-YF (**Figure 4E**). Ly49Q ITIM also has the potential to recruit SHIP1 and SHIP2 to the ruffled region (**Figures 4F, G**). These observations indicated that Ly49Q has the potential to affect actin dynamics by recruiting multiple actin-regulating proteins to its phosphorylated ITIM.

### Ly49Q binds sulfatide and modulates retention of cholesterol in the plasma membrane

Ly49Q localizes to lipid rafts and regulates their trafficking (13). Given that Ly49Q can recruit multiple actin remodeling molecules to lipid rafts and that lipid rafts are important in phagocytosis (47), it is important to determine whether or not Ly49Q-mediated actin regulation during phagocytosis is lipid raft-dependent.

**Figure 5:**
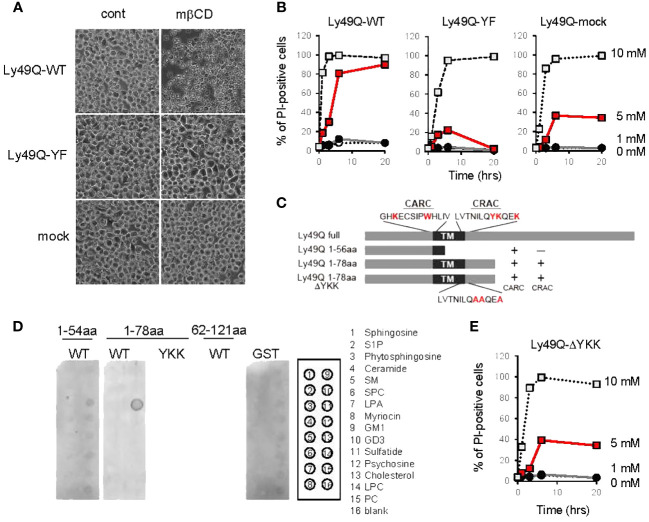
Regulation of membrane cholesterol by Ly49Q through its sulfatide binding ability. **(A)** Phase-contrast microscopy view of WEHI3 transfectants after treatment with 5 mM mβCD for 20 min. **(B)** WEHI3 transfectants were treated with different concentrations of mβCD as indicated for the indicated periods. The frequencies of PI-positive cells were examined by flow cytometry. **(C)** Schematic view of GST-fusion proteins prepared for analyzing the lipid binding ability of Ly49Q. The amino acids shown in red at the top represent the consensus motifs of CARC and CRAC, and YKK in the CRAC motif was substituted with AAA, as shown at the bottom. **(D)** The lipid binding ability of GST-fused Ly49Q was examined using Sphingo strips. Positions of lipid species spots are shown on the right. **(E)** The frequencies of PI-positive WEHI3 transfectant cells expressing the Ly49Q-ΔYKK mutant after treatment with mβCD were examined by flow cytometry. All results shown are representative of at least two separate experiments.

To this end, we treated Ly49Q transfectants (7) with methyl-β-cyclodextrin (mβCD) (22) to deplete cholesterol in order to disrupt lipid raft organization. Unexpectedly, treatment with 5 mM mβCD killed WEHI3 Ly49Q-WT cells in a short time (**Figures 5A, B**). WEHI3 Ly49Q-YF cells and mock transfectants were more resistant to this reagent than WEHI3 Ly49Q-WT cells (**Figures 5A, B**). Since cholesterol is required for the formation of the permeability barrier of the plasma membrane and mβCD acts strictly at the surface of the plasma membrane (48), these results suggest that acute cholesterol deprivation with mβCD may have caused leakage of the plasma membrane in WEHI3 Ly49Q-WT cells. These results also suggest that Ly49Q has some effect on cholesterol levels or localization in the plasma membrane.

To elucidate the mechanism underlying Ly49Q-dependent cholesterol regulation, we scrutinized Ly49Q’s primary structure and found two possible cholesterol binding motifs, CRAC and CARC, at lipid membrane boundary regions (**Figure 5C**, **Supplementary Figure 3A**) (30, 31). These cholesterol binding motifs were also identified in other Ly49 family members, and a typical sphingolipid-binding motif was also observed in some Ly49 family members, such as Ly49B and Ly49H (**Supplementary Figure 3A**).

We then examined the lipid binding ability of Ly49Q using GST-fused Ly49Q proteins (**Figure 5C**, **Supplementary Figure 3B**) but did not detect any binding of Ly49Q to cholesterol by liposome pull-down assays (**Supplementary Figure 3C**). Further examinations using protein-lipid overlay assays revealed that Ly49Q can bind sulfatide (**Figure 5D**). Ly49Q lacking CRAC (Ly49Q 1-56aa) or with substitution of the charged and hydrophilic amino acids in the CRAC motif (YKK) for AAA (Ly49Q 1-78aa ΔYKK) lost the ability to bind sulfatide (**Figures 5C, D**), indicating that Ly49Q binds sulfatide via the CRAC motif. Notably, WEHI3 cells expressing Ly49Q-ΔYKK were less sensitive to cholesterol depletion than Ly49A-WT (**Figures 5A, E**), indicating that the sulfatide binding ability of Ly49Q is critical for the retention of membrane cholesterol. A strong attractive force mediates the interaction between sulfatide and cholesterol to form a condensed structure in the lipid layer that mimics “lipid raft” domains (49).

Taken together, these observations strongly suggest that Ly49Q can self-organize lipid rafts and regulate intracellular cholesterol distribution by interacting with sulfatides via the CRAC motif.

### Ly49Q organized SM/GM1/cholesterol membrane domains in LAMP1-positive vesicles

**Figure 6:**
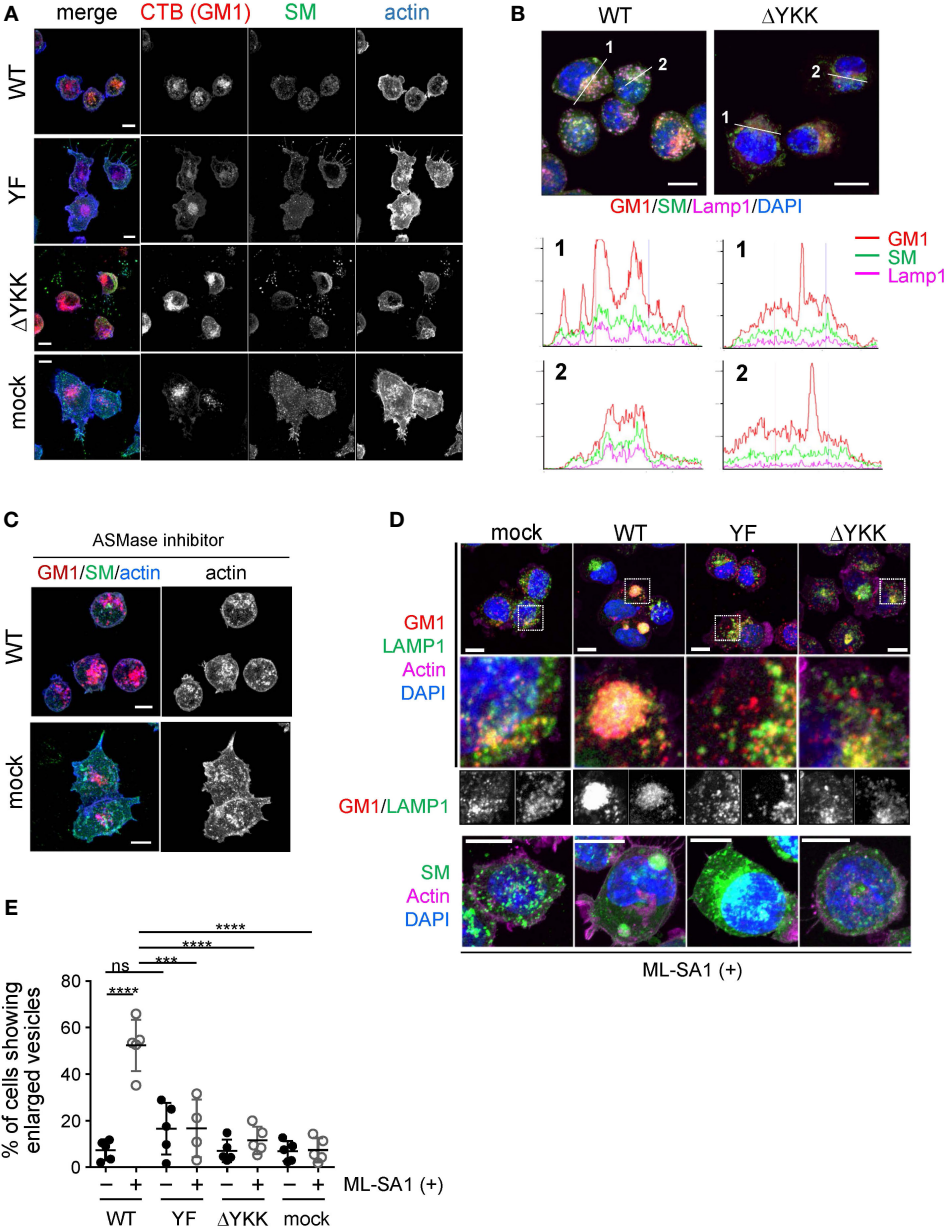
Significance of Ly49Q and its lipid binding ability in the regulation of sphingolipid distribution. **(A)** Intracellular distribution of sphingolipids in WEHI3 transfectants. GM1, SM, and F-actin were visualized using CTB (red), the SM probe, EquII (green), and phalloidin (blue), respectively. **(B)** Localization of sphingolipids in endolysosomes. WEHI3 transfectants expressing Ly49Q-WT or Ly49Q-ΔYKK were stained with CTB (red), EquII (green), anti-LAMP1 antibody (magenta), and DAPI (blue). Histograms show the fluorescence intensity profiles along the dashed lines in the upper photographs. **(C)** F-actin distribution in the presence of the AMSase inhibitor imipramine. WEHI3 transfectants were treated with imipramine (10 µM), and F-actin and sphingolipids were subsequently visualized as described above. Endolysosomal F-actin was detectable in WEHI3 Ly49Q-WT transfectants after treatment with imipramine. **(D)** Effects of Ly49Q on endolysosome behavior. WEHI3 transfectants were treated with ML-SA1, and the distribution of endolysosomes and sphingolipids was examined. Cells were stained as described in **(B)** after ML-SA1 treatment, and the areas within white boxes in the top photographs were enlarged. The bottom photographs are of cells stained with EquII (green) without CTB to clearly visualize SM localization together with counterstaining of F-actin (magenta) and DAPI (blue). **(E)** The frequencies of cells possessing SM-resident large LAMP1^+^ vesicles with or without ML-SA1 treatment. Scale bars: 10 µm. Statistical analyses were conducted using Mann–Whitney U test. ****P<0.0001; ***P<0.001; ns, not significant. All results shown are representative of at least two separate experiments.

To confirm the significance of the CRAC motif in Ly49Q-mediated lipid raft organization, we performed an immunohistochemical analysis by combining lipid-binding probes. SM and GM1 are the major constituents of lipid rafts and influence cholesterol distribution (50–52). We used FITC-labeled equinatoxin II (EquII) to detect SM (53) together with Alexa594-conjugated cholera toxin B subunit (CTB) to visualize lipid rafts (54). The distributions of both SM and GM1 differed substantially between WEHI3 Ly49Q-WT and other WEHI3 transfectants cells expressing ITIM-deficient or ΔYKK Ly49 mutants; SM colocalized with GM1 in vesicular compartments in WEHI3 Ly49Q-WT cells but not in any other WEHI3 transfectants (**Figure 6A**). SM in WEHI3 Ly49Q-WT cells was detected as small dots in the plasma membrane and cytosol, mostly together with GM1 (**Figure 6A**). The vesicles in which SM and GM1 colocalized were LAMP1^+^ vesicles (**Figure 6B**). Notably, in WEHI3 Ly49Q-YF and WEHI3 ΔYKK cells, SM patches were detected at the rear edge of filopodia, and the detachment and recovery of adhesion machinery appeared to have failed (**Figure 6A**); thus, colocalization of SM and GM1 in LAMP1^+^ granules was rarely detected (**Figures 6A, B**). Given that the ITIM of Ly49Q is needed for lipid raft trafficking (13), these results suggest that Ly49Q uses the CRAC motif to construct lipid rafts and places them in their proper locations via the ITIM.

Notably, WEHI3 cells expressing Ly49Q-YF, Ly49Q-ΔYKK, or control vector (mock) exhibited ectopic actin polymerization around intracellular granules and tips of filopodia, but WEHI Ly49Q-WT cells possessed little polymerized actin at these sites (**Figure 6A**). These results indicated that Ly49Q recruited SM to GM1^+^LAMP1^+^ granules and promoted actin depolymerization around these compartments in an ITIM- and CRAC motif-dependent manner. Importantly, the inhibition of acid sphingomyelinase (ASMase) by imipramine (55) resulted in actin polymerization around these granules (**Figure 6C**), strongly suggesting that an SM metabolite, such as ceramide, is involved in the regulation of actin polymerization in these LAMP1^+^ granules. Ceramide suppresses actin polymerization (56); therefore, Ly49Q-mediated SM compartmentalization in LAMP1^+^ granules may be involved in lysosomal actin dynamics via the ASMase function.

### Ly49Q-dependent membrane lipid arrangement in LAMP1^+^ granules affects granule behavior

LAMP1^+^ granules can be involved in phagocytic processes by supplying bulk membrane lipids to ruffling membranes and phagolysosomes via focal exocytosis (57, 58). Given that Mϕs with an impaired Ly49Q function were unable to form ruffling membranes and remodel actin properly, we hypothesized that Ly49Q might affect the behavior of LAMP1^+^ granules upon stimulation through its ITIM- and CRAC motif-dependent functions.

To test this hypothesis, we used a membrane-permeable mucolipin TRP channel 1 (TRPML1)-specific synthetic agonist, ML-SA1, which induces Ca^2+^ movement from the lysosome lumen into the cytosol (59). TRPML1 mediates Ca^2+^-dependent lysosomal trafficking and focal exocytosis, which supplies membrane lipids for phagosome formation (59). When WEHI3 transfectants were treated with ML-SA1, lysosomes in WEHI Ly49Q-WT cells gathered and coalesced into giant aggregates (**Figure 6D**). Such aggregated lysosome structures were hardly detectable in other transfectants of mock or mutant Ly49Q (**Figure 6D**). The frequency of cells showing aggregated lysosomes was significantly higher in WEHI Ly49Q-WT cells than in other transfectants (**Figure 6E**). Considering the function of TRPML, the aggregation of LAMP1^+^ granules in WEHI Ly49Q-WT cells may mimic the polarized movement of lysosomes and/or lysosome-related organelles (LROs) (59, 60). Although characterization of these aggregated LAMP1^+^ compartments is necessary, the above observations indicated that Ly49Q allocates SM and GM1 to LAMP1^+^ granules via its sulfatide-binding ability, which is critical for regulating LAMP1^+^ granule trafficking.

### Sulfatide is needed for phagocytosis with membrane ruffling and phagosome maturation

**Figure 7:**
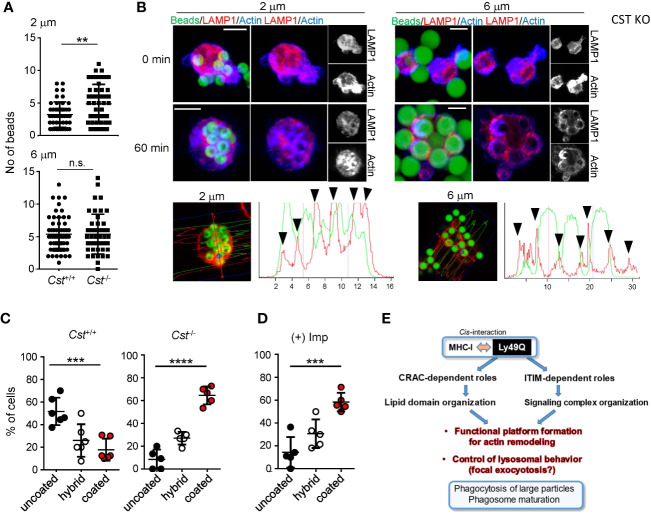
Significance of sulfatide in phagocytosis. PEMϕs were prepared from *Cst* ^+/+^ and *Cst* ^-/-^ mice, and phagocytosis of IgG-opsonized beads with different diameters was examined. **(A)** The numbers of ingested beads in single cells were counted. Each dot represents the number of beads ingested by a single cell. More than 30 cells were analyzed. **(B)** F-actin (blue) and endolysosomes (red) in PEMϕs that had ingested fluorescence-labeled beads (green) were visualized by staining with phalloidin and anti-LAMP1 antibody at the indicated time points. Histograms at the bottom show the fluorescence intensity profiles of beads (green) and F-actin (red) along the dashed lines in the left photographs. **(C, D)** Frequencies of cells possessing phagosomes with and without an actin coat in *Cst* ^+/+^ and *Cst* ^-/-^ PEMϕs **(C)** and in *Cst* ^+/+^ PEMϕs treated with imipramine (10 µM) **(D)**. Hybrid represents cells possessing both actin-coated and actin-uncoated phagosomes. All results shown are representative of at least two independent experiments. **(E)** Roles of Ly49Q in phagocytosis mediated by two different functional domains. Ly49Q has a CRAC-like motif and an ITIM; the former is needed for preparing functional membrane domains via its sulfatide binding ability, and the latter recruits a panel of signaling molecules to regulate actin remodeling and the distribution of Ly49Q together with the functional membrane domain. Regulation of both protein-lipid interactions by the CRAC motif and protein−protein interactions by the ITIM is needed for phagocytosis. Statistical analyses were conducted using Mann–Whitney U test **(A)** and one-way ANOVA **(C, D)**. ****P<0.0001; ***P<0.001; **P<0.01; ns, not significant. All results shown are representative of at least two separate experiments.

We finally confirmed the significance of sulfatide in phagocytosis and phagosome maturation using cerebroside sulfotransferase (CST)-null (*Cst* ^–/-^) mice (61). CST catalyzes the transfer of a sulfonate group from 3′-phosphoadenosine-5′-phosphosulfate (PAPS) to the C3 position of the nonreducing terminal galactose of glycolipid oligosaccharides to produce sulfatides and seminolipids (61, 62). *Cst* ^–/-^ mice therefore lack sulfatides and seminolipids (61). PEMϕs from *Cst* ^–/-^ mice exhibited no defects in the uptake of opsonized beads, irrespective of bead diameter, and exhibited a slight increase in the uptake of 2-µm beads (**Figure 7A**). Membrane ruffling after bead capture was barely detected in *Cst* ^-/-^ PEMϕs, and only cytoplasmic protrusions were observed (**Figure 7B**). Phagosome actin-coats in *Cst* ^-/-^ PEMϕs were preserved after incubation for 60 min (**Figure 7B**), and the actin coats around phagosomes were reduced in *Cst* ^+/+^ PEMϕs at the same time point (**Figure 7B**).

Similar results were obtained when BM-derived Mϕs (BMMϕs) were used. Approximately half of the *Cst* ^+/+^ BMMϕs showed actin-uncoated mature phagosomes, while the *Cst* ^-/-^ BMMϕs showed a decreased number of mature phagosomes and an increased number of actin-coated immature phagosomes (**Figure 7C**). These results indicated that sulfatide is needed for phagosome actin remodeling. Actin depolymerization of phagosomes was also ASMase-dependent in the BMMϕs because imipramine treatment of WT BMMϕs increased the ratio of cells possessing actin-coated phagosomes (**Figure 7D**), indicating that not only cell lines but also primary Mϕs use the SM-ceramide-axis to control phagosomal actin.

## Discussion

We revealed that an atypical and ancestral NK cell receptor, Ly49Q, confers a particular phagocytic effect on Mϕs and is needed for subsequent actin remodeling in phagosomes (**Figure 7E**). This may be important for phagosome maturation, as Ly49Q-deficient Mϕs showed reduced bactericidal activity against *Salmonella* infection. During phagocytic processes, Ly49Q regulates actin remodeling at the site of phagocytosis and around phagosomes, probably by interacting with MHC-I in *cis*. In addition to MHC-I binding, two functionally different Ly49Q motifs, the ITIM and the CRAC motif, play critical roles in phagocytosis; the latter is associated with sulfatide and organizes cholesterol- and SM-rich lipid raft domains, and the former recruits a panel of proteins involved in actin remodeling and places them together with lipid rafts to a proper intracellular site, such as LAMP1^+^ granules, which are the source of membrane lipids for promptly increasing membrane capacity via focal exocytosis during phagocytosis. When Mϕs engulf larger particles, they require an increased membrane capacity, including ruffled membrane formation, to encapsulate these large particles. Sphingolipids, such as sulfatide and SM, have saturated fatty acids and preferentially self-organize liquid-ordered membrane domains together with cholesterol (63). Ly49Q distributes to liquid-ordered lipid rafts via the CRAC motif and can assemble a panel of signaling molecules to lipid rafts via the ITIM domain.

Given that Ly49Q alters raft distribution in a stimulation-dependent manner (13), Ly49Q bearing both ITIM and CRAC motifs has the benefit of redistributing signaling complexes together with organized membrane lipids depending on inflammatory circumstances. Ly49Q-organized lipid rafts constitute membrane ruffles at the site of phagocytosis, and this membrane domain might subsequently be internalized with prey during phagocytosis. Thus, these membrane domains are thought to be primarily involved in the construction of the early phagosome membrane. In this context, actin in phagosomes and ruffled membranes is differentially regulated by the function of Ly49Q; Ly49Q promotes actin polymerization in membrane ruffles at the site of phagocytosis, while it promotes actin depolymerization at phagosomes. When promoting actin depolymerization, Ly49Q allocates SM to phagosomes, which enables local production of metabolites, such as ceramide, to regulate actin dynamics (64). Our findings also revealed that the allocation of an appropriate membrane lipid species to appropriate organelle membranes is controlled by membrane-spanning proteins and is critical for site-specific regulation of biological processes mediated by lipid metabolites.

Our observations indicate that Ly49Q exerts its functions during phagocytosis by interacting with MHC-I, probably in a *cis* configuration. Given that MHC-I clusters in the plasma membrane (65, 66) and considering the structural basis of MHC-I-Ly49 family interactions, in which one MHC-I molecule can possess more than one Ly49 binding site, i.e. sites 1 and 2, depending on context (67, 68), it is possible that the constitutive *cis*-interaction between Ly49Q and MHC-I can form a protein complex that includes multiple MHC-I molecules and Ly49Q homodimers. In addition to such stoichiometric assessments of protein−protein interactions, given that sphingolipid, as a saturated fatty acid, can organize a liquid-ordered domain by clustering (63, 69), *cis*-interaction between MHC-I and Ly49Q might incubate multivalent clusters of ITIMs within a limited membrane area. This might promote the formation of a membrane region where a panel of ITIM-interacting molecules are in close proximity to facilitate the construction of a functional lipid raft as a signaling platform for efficient and concerted regulation of complex biological processes, such as highly regulated actin remodeling. Given that Ly49Q expression is strongly induced by IFNγ in Mϕs (7), it is plausible that inflammatory stimulation might substantially affect the state of functional membrane domains, which strengthens the phagocytic potential and bactericidal activity of Mϕs.

Ly49Q-dependent organization of lipid rafts in LAMP1^+^ granules in WEHI3 cells allows for several interesting perspectives. LAMP1^+^ granules generally include late endosomes, lysosomes, and LROs, including cytotoxic granules and secretory lysosomes (70–72). The type of LAMP1^+^ granule in WEHI3 cells is currently unidentified; therefore, it is difficult to discuss Ly49Q-organized raft behavior in phagocytosis by superimposing the properties of WEHI3 LAMP1^+^ granules. However, because WEHI3 cells do not actively perform phagocytosis in the steady state, these vesicles in WEHI3 cells are nonphagosomes and are thus probably late endosomes or a certain type of LRO. Based on this scenario, it is intriguing to consider the source of membrane ruffles in the context of focal exocytosis, which is a mechanism for supplying bulk membrane lipids to the site of phagocytosis (59). We currently hypothesize that Ly49Q also mediates the focal exocytosis of lysosomes/LROs prior to making phagocytic membrane ruffles by organizing lipid raft domains in LAMP1^+^ granules. Our observation that the forced activation of TRPL channels induced trafficking of LAMP1^+^ granules in an Ly49Q-dependent manner suggested the possible involvement of Ly49Q in Ca^2+^-triggered lysosome secretion in a particular context and seems to support the above notion. In this context, it is intriguing to compare the LROs of Mϕs as the source of membrane ruffles with the LROs of NK cells, i.e. cytotoxic granules, both of which underlie focal exocytosis (73–75). It should be emphasized that other Ly49 family members also possess lipid binding motifs at the membrane boundary region. Given that Ly49Q is the ancestral gene of the Ly49 family, it would be interesting to consider the mechanistic similarity between prey capture by Mϕ phagocytosis and conjugation of NK cells with target cells (76).

NK cells also make Rho family-dependent ruffles when they attach to target cells, and impaired membrane ruffling severely decreases their cytotoxic activity (77). It is possible that the function of Ly49Q in ruffle formation may be diverted to other NK cell receptors possessing lipid-interacting motifs. According to this scenario, it is interesting to consider that NK cells probably control at least two different types of secretion machineries during their cytotoxic responses: one to supply bulk membrane lipids to make ruffled membranes, and the other for cytotoxic granule secretion. Lysosomes and lysosome-related organelles are highly heterogeneous compartments (60, 70); therefore, it is possible that different secretory pathways in NK cells may be differentially regulated by *cis*- and *trans*-interacting NK cell receptors. Alternatively, using membrane lipids supplied by the release of cytotoxic granules regulated by *trans*-interacting NK cell receptors, *cis*-interactable NK cell receptors may mediate ruffled membrane formation, thereby capturing the target. Although further precise investigations are needed to confirm these issues, our findings provide a novel perspective on the function of *cis*-interacting NK cell receptors.

Our data clearly demonstrate that Ly49Q provides a specialized and functionally important membrane domain in Mϕs through the integrative organization of protein−protein and protein-lipid interactions. There is still much to discover concerning the regulation of membrane architecture, including lipid rafts, as cells perform their various functions. Our results clearly demonstrate that membrane-spanning receptors precisely organize functional and cell type-specific membrane lipid environments. This mechanism may be critical for immune cells to perform their diverse functions.

## Materials and methods

### Mouse models

C57BL/6 *Klra17^–/–^* mice (13) and C57BL/6 *Cst* ^-/-^ mice (61) were described previously. C57BL/6J mice were purchased from CLEA Japan, Inc (Tokyo, Japan). All animal experiments were approved by the Animal Care and Use Committee of the National Center for Global Health and Medicine (NCGM) Research Institute and were conducted according to institutional procedures (No. 20050).

### Cells and antibodies

WEHI3 and its transfectants, except for WEHI3 Ly49Q-ΔYKK, were described previously (7, 13). WEHI3 Ly49Q-ΔYKK was established by substitution of tyrosine 71 and lysines 72 and 75 with alanines by polymerase chain reaction (PCR)-based cloning using PrimeSTAR GXL DNA polymerase (TaKaRa Bio Inc., Shiga, Japan) and the following primer set: Ly49Q Y71A K72A K75A, F 5′-CTTCTGGTAATTGTTGCAGTGTTGGTGACAAACATTTTGCAGGCTGCCCAAGAAGCCCATGAACTGCAGGAAAC-3′; Ly49Q WT R, 5′-AAGGACACAGAGGATTCCAAAAGCTATCACAATGAGATGCCAGGGGATAGAGCACTCTTTGTGGCCAGCTTCTC-3′.

PEMϕs were collected from the peritoneal cavity by injecting cold phosphate-buffered saline (PBS) containing 0.05% EDTA. Cells were cultured in RPMI 1640 Complete Medium (Nacalai Tesque Co. Kyoto, Japan) supplemented with 10% (v/v) fetal bovine serum (FBS), 10 mM 2-mercaptoethanol, 2 mM L-glutamine, 1 mM sodium pyruvate, 2 mM nonessential amino acids, 1% penicillin G/streptomycin, and 10 mM HEPES (pH 7.0) in a humidified incubator at 37°C with 5% CO_2_.

Bone marrow (BM)-derived Mϕs (BMMϕs) were differentiated from BM mononuclear cells in the presence of murine M-CSF (50 ng/mL) as described previously (78). Antibodies against the following proteins were purchased: phosphorylated ERK, ERK, phosphorylated IκBα, IκB, PLCγ2, Csk, Lyn, Shc, CrkL, Grb2, and β-actin from Cell Signaling Technology; LAMP1 (1D4B) from eBioScience; CD18 (M18/2) from BD Pharmingen; H-2Kb from BioLegend; Flag (M2) from Sigma; Acti-stain 670 Phalloidin from Cytoskeleton; Alexa Fluor 488-conjugated and Alexa Fluor 594-conjugated anti-Rabbit IgG, Alexa Fluor 594-conjugated anti-Mouse IgG, Alexa Fluor 594-conjugated anti-Rat IgG, Alexa Flour 594-conjugated phalloidin and Alexa Fluor 594-conjugated Cholera Toxin subunit B from Thermo Fisher Scientific; and Filipin III from Cayman Chemical Co.

### Phagocytosis assays

PEMϕs were seeded on fibronectin-coated round glass coverslips (ϕ12 µm; Fisher Scientific Inc. in 24-well plates and cultured for 4 h. In some experiments, PEMϕs were pretreated with imipramine (10 µg/ml) at 37°C and 5% CO_2_ for 30 min. Polybead® Polystyrene Microspheres (Polyscience Inc.) were coated with mouse IgG (1 mg/ml in PBS) at room temperature for 1 h and added to PEMϕs. Subsequently, the plate was spun to attach beads and further incubated at 37°C and 5% CO_2_ for the indicated periods. The bead-to-PEMϕs ratio was 1:5 for ϕ2-µm beads or 1:2 for ϕ6-µm beads. Two micrometer diameter beads were chosen in light of the fact that E. coli and yeast (Zymosan) are 2-4 and 3 micrometers, respectively. The larger ϕ6 μm beads were chosen because cellular debris is expected to be larger than E. coli or yeast. The erythrocytes physiologically phagocytosed by macrophages are 7 to 8 μm in size, and because in experimental practice phagocytosis efficiency decreases with bead size greater than ϕ10 μm, ϕ6 μm beads were chosen for quantitative accuracy. Cells were fixed with 4% paraformaldehyde (PFA), and immunohistochemical analyses were performed. When phagocytosed beads were counted, fluorescein isothiocyanate-conjugated microspheres were used, and surface binding was discriminated by staining with Alexa Fluor 594-conjugated anti-mouse IgG without cell permeabilization. For quantification of phagocytosis, 5 to 7 images of random fields were recorded, and the number of phagocytosed beads was counted in at least 30 cells. Phagocyte activity is represented as beads/cell.

### *S. typhimurium* infection *in vitro*

*S. typhimurium* strain SH100 WT or ΔInvA was used for *in vivo* infection experiments (33). PEMϕs were seeded into 24-well plates at a density of 5 × 10^5^ cells/well and infected with mouse serum-opsonized *S. typhimurium* with a multiplicity of infection of 5. Thirty minutes post infection, the culture medium was replaced with medium containing 100 μg/ml gentamicin for 1 h, and the gentamicin concentration was subsequently reduced to 10 µg/ml. The cells were further incubated for the indicated periods. Subsequently, cells were lysed with 1% Triton X-100 in PBS, and serially diluted cell lysates were plated onto LB agar. The plates were incubated at 37°C overnight, and the resulting colonies were counted. Cell death after *S. typhimurium* infection was assessed using an LDH-Cytotoxic Test Wako (FUJIFILM Co., Tokyo, Japan) according to the manufacturer’s instructions.

### *S. typhimurium* infection in mice

*S. typhimurium* strain SH100 WT was used for *in vivo* infection experiments. *S. typhimurium* (1 × 10^5^ cfu/mouse) was suspended in 100 µl PBS and intraperitoneally injected. Two days post-infection, the mice were sacrificed, and the spleens were weighed. To determine colony forming unit (CFU) in organs of infected mice, spleens and livers were homogenized using a Multibeads shocker cell disruptor (YASUI KIKAI, Osaka, Japan), and serial dilutions of homogenates were prepared and plated onto LB plates. The plates were incubated at 37°C overnight, and the resulting colonies were counted.

### Cytokine quantification

PEMϕs were infected with the *S. typhimurium* SH100 strain as described above, and their culture supernatants were collected at the indicated time points. IL6 levels in PEMϕ supernatants were measured using the Mouse ELISA Max Kit (BioLegend) according to the manufacturer’s instructions.

### Lipid binding assays

The lipid binding ability of Ly49Q was examined using Sphingo Strips (Echelon Biosciences Inc.) according to the manufacturer’s instructions. In brief, Shingo Strip membranes were incubated with GST-tagged Ly49Q proteins or control GST [5 µg/ml in tris-buffered saline (TBS) containing 3% bovine serum albumin (BSA)] at 4°C overnight. After washing with TBS-T to remove unbound GST-tagged proteins, Shingo Strip membranes were further incubated with anti-GST antibody (Santa Cruz Biotechnology, Inc.) and subsequently HRP-conjugated anti-mouse IgG (Jackson Laboratory). HRP was detected with Supersignal West Femto or Supersignal West Pico chemiluminescent substrates with the LAS-3000 detection system (FUJIFILM Co., Tokyo, Japan).

### Analyses of Ly49Q-cholesterol interaction using liposomes

Liposomes containing 90% phosphatidyl choline and 10% phosphatidyl serine with or without cholesterol (60%) were prepared as described previously (79). Liposomes were mixed with GST-tagged Ly49Q (5–7 mg/50 ml liposomes) and incubated at room temperature for 15 min. Subsequently, the liposomes were pelleted by centrifugation in a TLS-55 rotor (BECKMAN COULTER Co.) at 20°C for 30 min at 48000 rpm (200000 g). The supernatant (S) containing the free protein that was not bound to liposomes was collected. The liposome pellets (P) containing the cosedimented proteins were analyzed by sodium dodecyl sulfate-polyacrylamide gel electrophoresis (SDS−PAGE) followed by Coomassie brilliant blue staining or Western blotting using an anti-GST antibody.

### Protein expression in *Escherichia coli*

Plasmids encoding the SH2 domains of the phospho-ITIM-interacting proteins were introduced into BL21 (DE3) Rosetta cells, and protein expression was induced by incubation with 0.2 mM IPTG for 3 h at 30°C. Soluble fractions of BL21 Rosetta cells were prepared and used for peptide pull-down assays. GST-tagged Ly49Q deletion mutant proteins were collected from BL21 Rosetta cell soluble fractions using glutathione-Sepharose 4B columns according to the manufacturer’s instructions (Thermo Fisher Scientific) and eluted with 10 mM reduced glutathione in 50 mM Tris pH 8.0. Purified proteins were dialyzed overnight against PBS before use in lipid binding assays.

### Plasmid construction

To express the Glutathione S-Transferase (GST)-tagged Ly49Q mutants in *E. coli*, DNA corresponding to the mutant proteins indicated was amplified by PCR and cloned and inserted into pGEX4T1 (GE Healthcare Technologies Inc.). To express GST- or His_6_-tagged fusion proteins in *E. coli*, DNA corresponding to the SH2 domains of the phospho-ITIM-interacting proteins was amplified by PCR and cloned and inserted into pGEX4T1 or pET19b (Merck Millipore Co.). The expressed SH2 domains were as follows: mouse CrkL (residues 1–110), mouse Lyn (residues 101–250), mouse Shc (residues 451–579), mouse Grb2 (residues 41–190), mouse Csk (residues 50–200), mouse Hck (residues 100–250), mouse Fgr (residues 100–250), and mouse PLCγ2 (residues 501–640, 501–750, and 625–750). Mouse Grb2 and Ly49Q were cloned and inserted into pCAG-HA or pCAGGS-EGFP expression vectors.

### Peptide pull-down assays

Peptides corresponding to the tyrosine-phosphorylated ITIM of Ly49Q (CGGMSEQEVTpYSTVRFHK) and to the nonphosphorylated ITIM (CGGMSEQEVTYSTVRFHK) were synthesized by Operon Biotechnologies Inc.) and conjugated to SulfoLink resin according to the manufacturer’s instructions (Thermo Fisher Scientific). Lysates of Raw264.7 cells were prepared with lysis buffer containing 50 mM Tris-HCl (pH 7.5), 1 mM EDTA, 10% glycerol, 150 mM NaCl, 1% NP-40, and protease/phosphatase inhibitors (Thermo Fisher Scientific) and incubated with the peptide-conjugate resins for 1 h at 4°C. The resins were then washed with lysis buffer, and the proteins bound to the resin were separated by SDS−PAGE and visualized by silver staining. Proteins specifically bound to the phosphorylated ITIM peptides were excised and subjected to liquid chromatography-tandem mass spectrometry (LC−MS/MS; APRO Science Inc.).

### Immunoprecipitation

Cell lysates of WEHI3 cells were prepared with lysis buffer containing 50 mM Tris-HCl (pH 7.5), 1 mM EDTA, 10% glycerol, 150 mM NaCl, 1% Triton-X100, and protease/phosphatase inhibitors. In some experiments, the cells were subjected to pervanadate treatment prior to preparing the lysates. The obtained cell extracts were incubated with antibody-conjugated agarose beads for 1 h at 4°C, and the precipitated proteins were analyzed by SDS−PAGE and immunoblotting.

### Transfection of RAW264.7 cells

RAW264.7 cells were transfected with GFP-tagged Ly49Q-WT or GFP-tagged Ly49Q-YF expression vectors using the Neon transfection system according to the manufacturer’s instructions (Life Technologies Inc.). HEK293T cells were transfected with Myc-tagged SHIP1 or SHIP2 expression vectors using Fugene HD (Roche Inc.).

### Time-lapse analyses

PEMϕs were seeded in glass-bottom culture dishes (CELLview™ Cell culture dish; Greiner Bio-One Co.) and cultured overnight. Time-lapse images of PEMϕs were acquired using a BZ-X710 microscope (Keyence, Osaka, Japan) equipped with a CFI60 *CFI Plan Apoλ 60× objective* (Nikon Co., Tokyo, Japan).

### Biochemical experiments

WEHI3 transfectants were lysed in 1% NP-40 lysis buffer (50 mM Tris-HCl [pH 7.5], 1 mM EDTA, 10% glycerol, 150 mM NaCl, 1% NP-40) containing Halt protease inhibitor cocktail and Halt phosphatase inhibitor cocktail EDTA-free (Thermo Fisher Scientific). After extraction by rotation at 4°C for 30 min, cell lysates were collected by centrifugation, and anti-Flag antibody (M2; Sigma) was added. After incubation on ice for 1 h, the cell lysates were further incubated with rabbit anti-mouse IgG and Protein G beads at 4°C with rotation for 1 h. After washing the beads 3 times with lysis buffer, the precipitates were collected in SDS-sample buffer, heated to 95°C for 5 min, and analyzed by Western blotting.

### Cholesterol depletion experiments

WEHI3 transfectants in 24-well plates were treated with the indicated concentration of methyl-β-cyclodextrin (Sigma-Aldrich) for the indicated periods. The cells were collected in propidium iodide (PI) (Sigma)-containing FACS buffer (PBS containing 0.1% BSA and 0.5% EDTA) and immediately analyzed by flow cytometry (FACSCalibur; Becton Dickinson and Company Co.). The percentages of PI-positive cells were analyzed by CellQuest (FACSCalibur; Becton Dickinson and Company Co.).

### Gene expression analyses

RNA from cells was purified using an RNeasy Mini Kit (QIAGEN Inc., Germany) or ISOGEN (NIPPON GENE Co., Tokyo, Japan) and reverse-transcribed using the LunaScript™ RT SuperMix Kit (New England Biolabs Inc.). SYBR-based quantitative PCR was conducted with the StepOne Plus Real-Time PCR System (Thermo Fisher Scientific) with specific primers for murine IL6 or HPRT (Thermo Fisher Scientific). Gene expression was normalized to HPRT.

### Statistical analyses

The statistical significance of differences between obtained values was determined by an analysis of variance, the Mann−Whitney U test or One-way ANOVA using the Prism 6.0 software program (GraphPad Software Inc.). Data are presented as mean ± standard deviation (SD). Differences with a *P* value <0.05 were considered significant.

## Data availability statement

The raw data supporting the conclusions of this article will be made available by the authors, without undue reservation.

## Ethics statement

The animal study was approved by The Animal Care and Use Committee of the National Center for Global Health and Medicine (NCGM) Research Institute. The study was conducted in accordance with the local legislation and institutional requirements.

## Author contributions

HK: Investigation, Writing – review & editing. TN: Resources, Writing – review & editing. YS: Investigation, Validation, Writing – review & editing. SH: Investigation, Writing – review & editing. SS: Investigation, Writing – review & editing. TH: Methodology, Resources, Writing – review & editing. KM: Investigation, Methodology, Resources, Writing – review & editing. KK: Resources, Writing – review & editing. KT: Investigation, Supervision, Writing – review & editing. NG: Supervision, Writing – review & editing. KH: Methodology, Resources, Writing – review & editing. TT: Investigation, Methodology, Resources, Writing – review & editing. HS: Data curation, Supervision, Writing – review & editing. NT: Conceptualization, Data curation, Formal analysis, Funding acquisition, Project administration, Supervision, Validation, Visualization, Writing – original draft, Writing – review & editing.

## Supplementary material

Supplementary Figure 1 Involvement of Ly49Q in the responsiveness of Mϕs to stimuli. **(A)** Time lapse analyses of PMA-stimulated PEMϕs obtained from *Klra17^+/+^* and *Klra17^-/-^* mice. **(B)** Photographs show phase-contrast images of PEMϕs at the indicated time points in the time lapse movie shown in **(A)**. **(C, D)** Cell responses of *Salmonella*-infected PEMϕs were analyzed. **(C)** NF-κB activation by *Salmonella* in PEMϕ. Total cell lysates from *Salmonella*-infected PEMϕs were analyzed by western blotting using the antibodies indicated. **(D)** *Salmonella*-induced IL6 secretion by PEMϕs was evaluated by ELISA. Statistical analyses were conducted using Mann–Whitney U test. *P<0.05.

Supplementary Figure 2 Phagocytosis of IgG-opsonized latex beads in PEMϕs expressing Ly49Q-WT or Ly49Q-YF. **(A)** Each dot represents the number of ingested beads in a single cell. More than 30 cells were examined. Statistical analyses were conducted using Mann–Whitney U test. ns, not significant. **(B)** Optical view of bead-bound (left) and bead-ingested (right) PEMϕs. Surface-bound beads were discriminated by staining with Alexa Fluor 594-conjugated anti-mouse IgG antibody without cell permeabilization. **(C-E)** Serial sections of the Z-plane at the site of phagocytosis shown in **Figure 2B** **(C)**, **Figure 2C** **(D)**, and **Figure 2D** **(E)**. Arrowheads in **(D)** show endosomal polymerized actin.

Supplementary Figure 3 Potential binding of Ly49 to sphingolipids. **(A)** Possible lipid binding motifs in Ly49 family members. Lipid binding motifs, including CRAC (red) and sphingolipid-binding motif (green), are indicated in the amino acid alignment of Ly49 family members. ITIM is indicated in blue. **(B)** Preparation of GST-fused Ly49Q fragments. GST-fused Ly49Q was expressed in *E. coli* as described in the Methods, and total lysates were analyzed for the expression of GST-fused proteins (red arrows). Proteins were visualized by Coomassie brilliant blue staining. WCL; whole cell lysate, PD; pull-down. **(C)** Evaluation of the cholesterol binding abilities of Ly49Q and MHC-I using cholesterol-containing liposomes. GST fusion proteins of full-length Ly49Q or H-2Kb were mixed with cholesterol-containing liposomes, and proteins that coprecipitated with liposomes were analyzed by SDS−PAGE followed by western blotting using an anti-GST antibody. S; supernatant, P; precipitate.

Supplementary Movie 1 Time lapse movie of PMA-stimulated PEMϕs obtained from *Klra17^+/+^* mice. *Klra17^+/+^* PEMϕs showed active membrane ruffling under PMA-stimulation.

Supplementary Movie 2 Time lapse movie of PMA-stimulated PEMϕs obtained from *Klra17^-/-^* mice. Ruffled membrane formation induced by PMA-stimulation was impaired in *Klra17^-/-^* PEMϕs.
