# Supplementary figures and images for: Collaboration between a cis-interacting natural killer cell receptor and membrane sphingolipid is critical for the phagocyte function
Source: Front Immunol. 2024 Apr 24;15:1401294. doi: 10.3389/fimmu.2024.1401294 (PMC11076679; doi:10.3389/fimmu.2024.1401294)

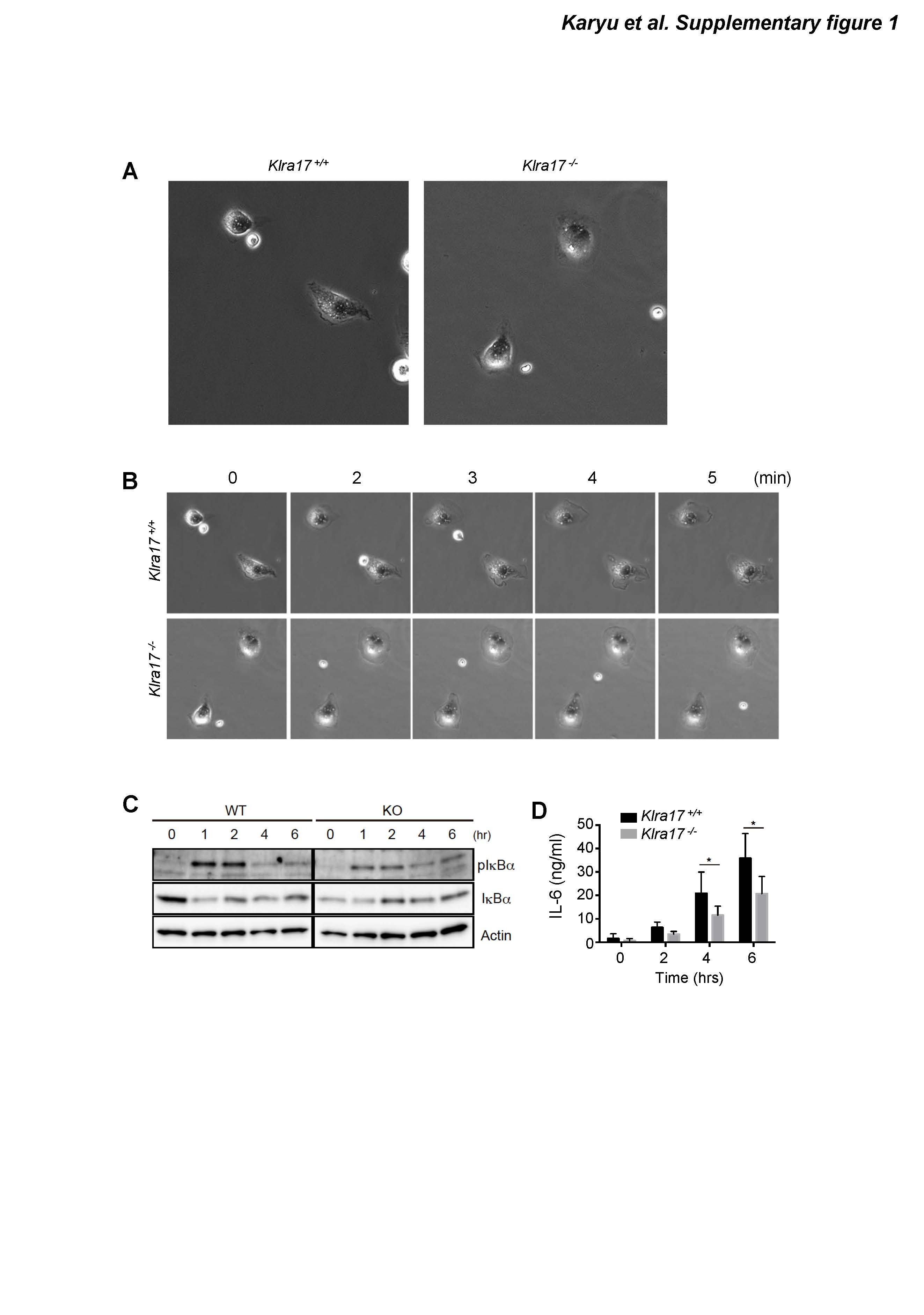

Supplement: Supplementary Figure 1 — Involvement of Ly49Q in the responsiveness of Mϕs to stimuli. (A) Time lapse analyses of PMA-stimulated PEMϕs obtained from Klra17+/+ and Klra17-/- mice. (B) Photographs show phase-contrast images of PEMϕs at the indicated time points in the time lapse movie shown in (A). (C, D) Cell responses of Salmonella-infected PEMϕs were analyzed. (C) NF-κB activation by Salmonella in PEMϕ. Total cell lysates from Salmonella-infected PEMϕs were analyzed by western blotting using the antibodies indicated. (D) Salmonella-induced IL6 secretion by PEMϕs was evaluated by ELISA. Statistical analyses were conducted using Mann–Whitney U test. *P<0.05. [file Image_1.jpeg]

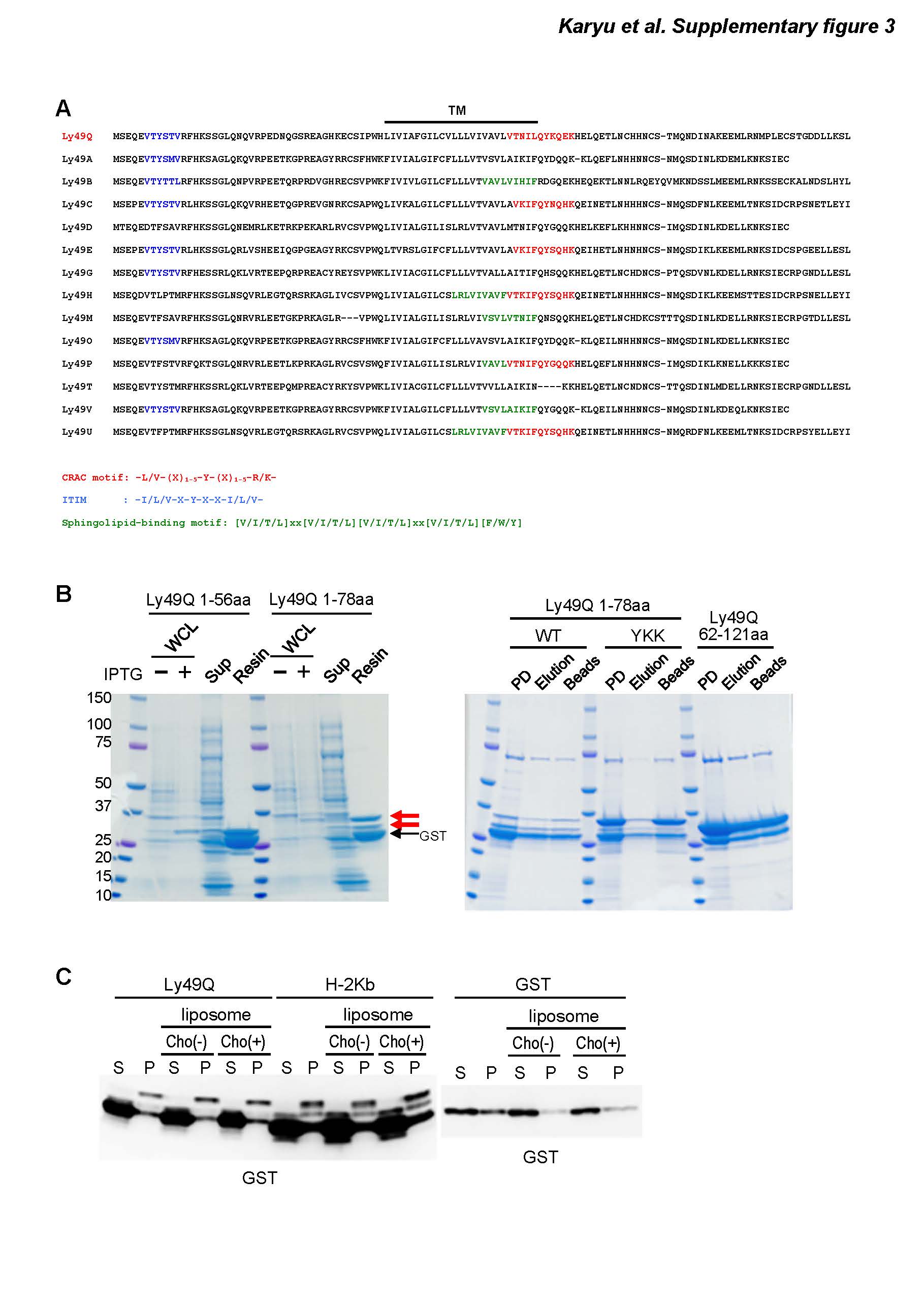

Supplement: Supplementary Figure 3 — Potential binding of Ly49 to sphingolipids. (A) Possible lipid binding motifs in Ly49 family members. Lipid binding motifs, including CRAC (red) and sphingolipid-binding motif (green), are indicated in the amino acid alignment of Ly49 family members. ITIM is indicated in blue. (B) Preparation of GST-fused Ly49Q fragments. GST-fused Ly49Q was expressed in E. coli as described in the Methods, and total lysates were analyzed for the expression of GST-fused proteins (red arrows). Proteins were visualized by Coomassie brilliant blue staining. WCL; whole cell lysate, PD; pull-down. (C) Evaluation of the cholesterol binding abilities of Ly49Q and MHC-I using cholesterol-containing liposomes. GST fusion proteins of full-length Ly49Q or H-2Kb were mixed with cholesterol-containing liposomes, and proteins that coprecipitated with liposomes were analyzed by SDS−PAGE followed by western blotting using an anti-GST antibody. S; supernatant, P; precipitate. [file Image_3.jpeg]
